# Undifferentiated colonic neoplasm with SMARCA4 germline gene mutation and loss of SMARCA4 protein expression: a case report and literature review

**DOI:** 10.1186/s13000-021-01091-6

**Published:** 2021-04-09

**Authors:** Huanli Duan, Wei Gao, Leiming Wang, Feng Cao, Lianghong Teng

**Affiliations:** 1 Department of Pathology, Xuanwu Hospital, Capital Medical University, 45 Changchun Street, Xicheng District, Beijing, China; 2 Department of General Surgery, Xuanwu Hospital, Capital Medical University, 45 Changchun Street, Xicheng District, Beijing, China

**Keywords:** Undifferentiated colonic carcinoma, SMARCA4, Germline mutation

## Abstract

**Background:**

Nonsense mutation or inactivation of SMARCA4 (BRG1) is associated with a monomorphic undifferentiated histological appearance in tumors at different sites. The association between SMARCA4 alteration and undifferentiated colonic carcinoma needs to be further elucidated.

**Methods:**

A 61-year-old male patient presented to the hospital with intermittent epigastric pain in the right upper abdomen and abdominal distension. The enhanced computed tomography detected a mass in the hepatic flexure of the colon and multiple liver metastases.

**Results:**

The right hemicolectomy contained a 4.5-cm undifferentiated malignancy with cells arranged in sheets, abundant necrosis, and areas showing rhabdoid morphology. The immunohistochemistry result showed that these tumor cells were focally positive for cytokeratin (CK), CK8, and CK18; however, diffusely positive for vimentin, P53, Fli-1, and SALL-4. Notably, tumor cells showed a heterogeneous loss of SMARCA4 expression pattern and intact SMARCB1 expression. Next-generation sequencing showed a germline SMARCA4 c.3277C>T(p.R1093*)mutation, somatic APC mutation, and no abnormal SMARCB1 gene. The tumor exhibited microsatellite stability, negative PD-L1 expression, and few infiltrating CD8 + T cells. The patient died a month later after surgery.

**Conclusions:**

We presented a rare case of undifferentiated colonic neoplasm with loss of SMARCA4 protein expression and germline SMARCA4 mutation. Moreover, the role of SMARCA4 alterations in tumor diagnosis and treatment was also summarized.

**Supplementary Information:**

The online version contains supplementary material available at 10.1186/s13000-021-01091-6.

## Background

The switch/sucrose non-fermenting (SWI/SNF) complex was first discovered in *S. cerevisiae* and revealed evolutionary conservation from yeast to mammals. It contains approximately 10–12 subunits. The SWI/SNF interacts with histones and transcription factors to modulate chromatin structure and control gene expression. The ATP-dependent chromatin remodeling complex is involved in regulating diverse biological functions, including differentiation and cell proliferation. Tumor suppressor SMARCA4 is a catalytic ATPase subunit of the SWI/SNF complex. Inactivation of SMARCA4 and other subunits of the SWI/SNF complex have been associated with a monomorphic undifferentiated histological appearance in tumors at different sites. SMARCA4 is frequently inactivated by mutations or other mechanisms in malignancies, such as non-small cell lung cancer, thoracic sarcoma, and malignant rhabdoid tumors [1–3]. SMARCA4 mutations (both somatic and germline mutations) are currently recognized as genetic driver events in almost all small cell carcinomas of the ovary, hypercalcemic type (SCCOHT), which is the most common undifferentiated ovarian malignancy in women under 40 years of age [4, 5]. Potential targeted therapies, such as anti-PD-1 antibodies and CDK4/6 inhibitors, have been reported for treating SMARCA4-deficient non-small cell lung cancer (SMARCA4-deficient NSCLC) [1, 6]. In the gastrointestinal tract, undifferentiated carcinomas are a rare highly aggressive malignancy with frequent rhabdoid features [5]. The association between undifferentiated carcinoma and SMARCA4 alterations, especially the genetic alterations, has not been elucidated. We reported a rare case of a 61-year-old man diagnosed with undifferentiated colonic neoplasm with a heterogeneous pattern of loss expression of SMARCA4 and germline SMARCA4 mutation, as well as predictive markers for potential immunotherapy or targeted therapy. Moreover, the role of SMARCA4 alterations in diagnosing and treating other tumors was also discussed.

## Case presentation

**Fig. 1:**
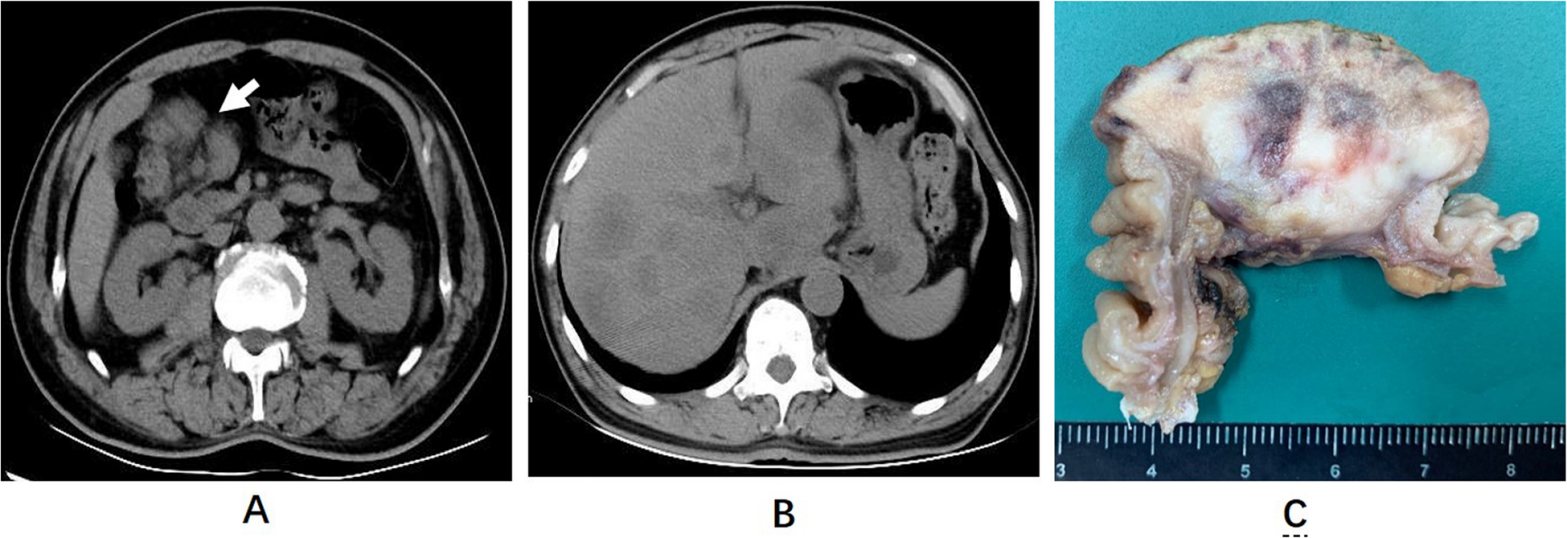
Axial CT scan at the level of the kidneys (**a**) and liver (**b**) demonstrates a mass (white arrow) within the hepatic flexure of the colon and multiple liver masses, respectively. The cut surface (**c**) shows the tumor has penetrated through the muscle wall of the colon, exhibiting fine texture with central hemorrhagic necrosis

**Fig. 2:**
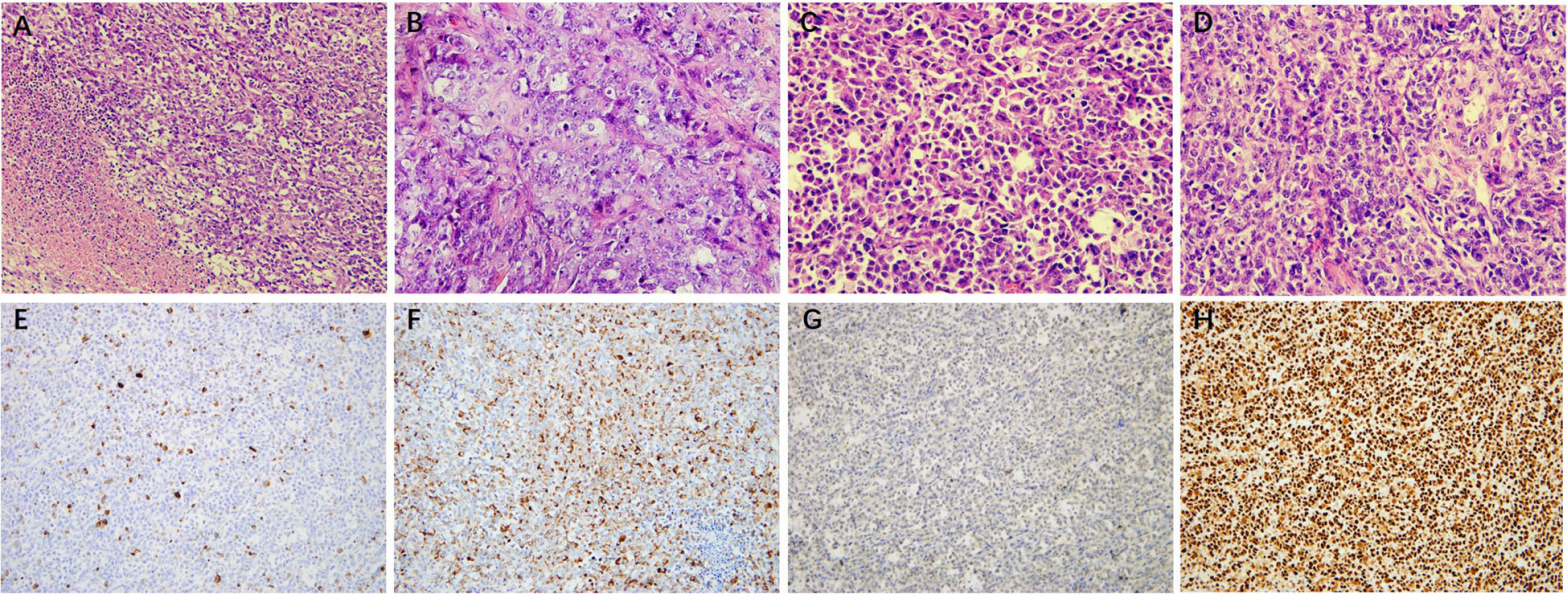
Hematoxylin and eosin staining show the tumor exhibited a sheet-like structure with necrosis (**a**), vesicular nuclei and prominent nucleoli (**b**) and areas with rhabdoid morphology (**c**). The tumor cells show a high mitotic rate (**d**). Tumor cells are positive for (**e**) CK and (**f**) vimentin. SMARCA4 (BRG1) (**g**) was lost, while SMARCA2 (INI-1) (**h**) was retained in the tumor cell nuclei

A 61-year-old man presented to the Xuanwu Hospital with intermittent epigastric pain in the right upper abdomen and abdominal distension. He had a history of gastric ulcer. The patient’s aunt previously died of colorectal cancer when she was 50 years old. An abdominal computed tomography (CT) scan revealed a mass in the right colon and multiple liver metastases (Fig. 1a, b). The patient subsequently underwent a partial colectomy. The right hemicolectomy revealed a 4.5-cm tumor invading through the muscularis propria into the subserosa (Fig. 1c). The cut surface showed a white to a reddish-brown tumor with central hemorrhage and necrosis. Histologically, the tumor exhibited a sheet-like structure composed of large cells with nucleoli. We also observed abundant necrosis and areas showing rhabdoid morphology (Fig. 2a-d). The immunohistochemistry result showed that these tumor cells were focally positive for cytokeratin (CK), CK8, and CK18; however, diffusely positive for vimentin, P53, Fli-1, and SALL-4. They were negative for CK20, CDX-2, Desmin, Myoglobin, MyoD1, Myogenin, CD56, Syn, and S-100. Notably, tumor cells showed loss of SMARCA4 protein and intact SMARCB1 protein expression (Fig. 2e-h). Next-generation sequencing (NGS) was performed, and the details are available in the Supplementary Material. NGS showed a germline SMARCA4 c.3277C>T(p.R1093*)mutation and no abnormal SMARCB1 gene. The tumor exhibited microsatellite stability (tumor mutation burden (TMB) of 1.68muts/Mb) and negative PD-L1 expression (tumor proportion score less than 1% and combined positive score less than 1) (Table 1).

**Table 1:** Next-generation sequencing analysis

Gene/Biomarker	Alteration (frequency)
SMARCA4	Germline mutation p.R1093*
APC	Somatic mutation p.R216*(31.46%)
JUN	Somatic mutation p.K311Sfs*6 (20.77%) & pN85Pfs*21 15.93%)
NRAS	Wild-type
KRAS	Wild-type
BRAF	Wild-type
PD-L1	Negative expression
TNM	1.68Muts/Mb
MSA	MSS
HLA-I	Heterozygous type

*TNM* tumor mutation burden, *MSA* microsatellite analysis, *MSS* microsatellite stability

The patient’s physical condition was poor, with multiple liver metastases and several serious complications, such as obstructive jaundice, ascites, anemia, and incomplete intestinal obstruction during hospitalization. The patient died a month later after surgery.

Published literature describes the clinicopathological and molecular features of SMARCA4-deficient tumors, which are summarized in Supplementary Table 1.

## Discussion and conclusions

SMARCA4 gene encodes a protein called BRG1, which is suggested to have a tumor suppressor role [5]. SMARCA4 inactivation has been identified as the main genetic driver event in several malignancies, such as SMARCA4-deficient undifferentiated uterine sarcoma, SCCOHT, SMARCA4-deficient thoracic sarcoma (SMARCA4-DTS), and SMARCA4-deficient NSCLC [4, 7–9]. Patients with SMARCA4-deficient neoplasms refer to high-grade malignancies associated with a dismal prognosis. Therefore, a diagnosis based on morphology and immunohistochemistry is particularly important.

SMARCA4-deficient neoplasms typically show an undifferentiated, often rhabdoid morphology and demonstrate a loss of nuclear staining for BRG1 by immunohistochemistry [10]. However, poorly differentiated and at least focal differentiated (glandular or squamous) phenotypes are also observed in SMARCA4-DTS and SMARCA4-deficient NSCLC, respectively [9, 11]. Currently, whether it is called SMARCA4-deficient sarcoma or SMARCA-deficient carcinoma remains controversial. Witkowski et al. questioned the current classification of SCCOHT. Despite the name of ‘carcinoma’, SCCOHT is classified as ‘miscellaneous ovarian tumors’ according to the 4th edition World Health Organization classification of ovary tumors [4]. Moreover, previous studies reported that loss of SMARCA4 expression caused tumor cells to undergo an epithelial-to-mesenchymal transition in lung adenocarcinoma and human mammary epithelial cells [12, 13]. Given the molecular and genetic features of SMARCA4-deficient neoplasms, the name ‘SMARCA4-deficient sarcoma’ might be more suitable. We present an undifferentiated colonic neoplasm with loss of SMARCA4 expression and germline SMARCA4 nonsense mutation where tumor cells were focally positive for CK, CK8, and CK18, but diffusely positive for vimentin.

Twenty-six percent of undifferentiated carcinomas showed loss of core SWI/SNF proteins in the digestive system, including SMARCA4, SMARCA2, SMARCB1, ARID1A, and ARID1B proteins. However, none of the poorly differentiated gastrointestinal carcinomas had a loss of core SWI/SNF detected proteins. SWI/SNF-deficient undifferentiated carcinomas exhibited a sheet-like growth pattern, with cellular decohesion and a rhabdoid appearance. The frequency of loss of SMARCA4 expression was lower than that of SMARCA2, which was the most common non-expressed protein among the five core SWI/SNF proteins. SMARCA4-deficient undifferentiated carcinoma was not common, often with an intact expression of SMARCB1 [5, 14].

Four types of altered SMARCA4 expression were identified in gastric cancer, exhibiting lost, reduced, heterogeneous pattern of lost, and heterogeneous pattern of reduced. These patients were mainly male (male: female = 2.1:1), and the median age was 70 years old (range 43–87). Inactivating mutations in the SMARCA4 gene led to the loss of the SMARCA4 protein. SMARCA4 mutations were detected mainly in SMARCA4-lost and heterogeneous pattern of SMARCA4-lost gastric cancer, while ARIDIA mutations mainly in SMARCA4-reduced and heterogenous pattern of SMARCA4-reduced gastric cancer [15], which is consistent with the finding report in the current study case. We showed that germline SMARCA4 mutations were detected in an undifferentiated colonic neoplasm with a heterogeneous pattern of SMARCA4 loss expression. Moreover, the patient’s aunt previously died of colorectal cancer when she was 50 years old. Ulrika A. Hänninen et al. reported that SMARCA4 mutations were detected by NGS in 6% of small bowel adenocarcinoma. However, SMARCA4 immunohistochemical stains were not performed, and data about the SMARCA4 protein expression is not available [16].

Somatic SMARCA4 mutations occur in lung adenocarcinomas, lymphomas, and medulloblastomas. They are associated with a poor prognosis [17]. In NSCLC, somatic SMARCA4 mutations were detected in 10% NSCLC. Forty-five percent of SMARCA4 mutant NSCLC reported the loss of SMARCA4 expression, and 90% had truncating SMARCA4 mutation. In other words, truncating SMARCA4 mutations are the key genetic event accounting for the SMARCA4-deficient NSCLC. Moreover, clinical outcomes are poor in this molecular subgroup [18, 19].

Rhabdoid Tumor Predisposition Syndrome (RTPS) are due to pathogenic variant in genes of SMARCB1 (PTRS1, commonly,) or SMARCA4(PTRS2, rarely), which are inherited in an autosomal dominant fashion. Patients with RTPS, especially RTPS1, are at increased risk to grow rare and highly aggressive rhabdoid tumor, predominantly in infants and children younger than 3 years old. RTPS2 exposes female carriers to an ill-defined risk of SCCOHT, which may appear in prepubertal females. The penetrance of RTPS1 may be over 90% by 5 years. However, the penetrance of RTPS2 was not well known. Similar to somatic SMARCA4 mutation, RTPS1 and RTPS2 are characterized by a predominance of truncating mutation [20]. The germline SMRACA4 mutation in the present study was also a truncating mutation. In SCCOHT, at least one germline or somatic deleterious SMARCA4 mutation was detected in 94% of cases. Although germline and somatic SMRACA4 mutations were detected in one familial case, germline SMRACA4 mutations were the key genetic event [4].

PD-1 inhibitors and CDK4/6 inhibitors were reported to be effective for treating certain SMARCA4-deficient neoplasms. Specifically, a patient diagnosed with SMARCA4-DTC showed a partial response with only one dose of Pembrolizumab. Sixty percent of the tumor cells expressed programmed cell death ligand-1 protein [21]. A patient with SMARCA4-deficient lung adenocarcinoma exhibiting a high tumor mutation burden was successfully treated with nivolumab [6]. In SMARCA4-deficient NSCLC, SMARCA4 loss results in reduced cyclin D1 expression and selective sensitivity to cyclin-dependent kinase 4/6(CDK4/6) inhibitors in vitro and in vivo, suggesting that FDA-approved CDK4/6 inhibitors could be effective for treating neoplasm [1]. Similarly, the immunochemical analysis of the present case indicated that the tumor cells were negative for CyclinD1. Furthermore, the tumor was PD-L1 negative and showed few infiltrating CD8+ T cells and a low tumor burden. Therefore, the patient may not benefit from the immune checkpoint inhibitors [22]. However, wild type of NRAS, KRAS, and BRAF genes suggest the tumor may be sensitive to endothelial growth factor receptor targeting antibody.

Somatic APC mutation p.R216* was also detected in 31% of tumor cells in this case. APC gene is widely accepted as a colorectal cancer tumor suppressor gene. Somatic APC mutations are detected in at least 80% of sporadic colorectal tumors [23].

We reported a rare case of a 61-year-old man who was diagnosed with undifferentiated colonic neoplasm with a heterogeneous pattern of loss expression of SMARCA4 and germline SMARCA4 mutation, as well as predictive markers for potential immunotherapy or targeted therapy. Moreover, the role of SMARCA4 alterations in diagnosing and treating other tumors was also summarized.

## Supplementary Information

**Additional file 1: Supplementary Table 1.** The clinicopathological and molecular features of SMARCA4-deficient tumors were summarized.

**Additional file 2.**

## Data Availability

All data generated or analyzed during this study are included in this article and its supplementary information files.

## Abbreviations

CK: Cytokeratin
SWI/SNF: Switch/sucrose non-fermenting
CT: Computed tomography
TMB: Tumor mutation burden
MSS: Microsatellite stability
MSA: Microsatellite analysis
SCCOHT: SMARCA4-deficient undifferentiated uterine sarcoma, small cell carcinoma of the ovary, hypercalcemic type
SMARCA4-DTS: SMARCA4-deficient thoracic sarcoma
SMARCA4-deficient NSCLC: SMARCA4-deficient non-small cell lung cancer
CDK4/6: Cyclin-dependent kinase 4/6
RTPS: Rhabdoid Tumor Predisposition Syndrome
