# Supplementary material for: Undifferentiated colonic neoplasm with SMARCA4 germline gene mutation and loss of SMARCA4 protein expression: a case report and literature review
Source: Diagn Pathol. 2021 Apr 9;16:30. doi: 10.1186/s13000-021-01091-6 (PMC8033741; doi:10.1186/s13000-021-01091-6)
Supplement: Supplementary file 1 — Additional file 1: Supplementary Table 1. The clinicopathological and molecular features of SMARCA4-deficient tumors were summarized. [file 13000_2021_1091_MOESM1_ESM.pdf]

Supplementary Table 1. The clinicopathological and molecular features of SMARCA4-deficient tumors were summarized.

References	Pathology	Median age	Morphology	IHC	Molecular change	Prognosis	Potential treatment
Kolin, D. L. 2018	SDUS	36 year old	sheets of large atypical epithelioid cells with prominent rhabdoid morphology	Loss of SMARCA4, negative of focally positive for keratins, EMA	SMARCA4 mutation	Extensive lymphovascular invasion; extra-uterine spread	
Douglas I. L,2019	SDUS	49 year old	predominantly rhabdoid or large epithelioid cells with abundant cytoplasm, but also had varying degrees of small cell and spindle cell morphology.	Loss of SMARCA4	Germline SMRCA4 mutation, MSS	Most tumors were aggressive with distant metastasis	
Raul P,2019 and Takada K 2019	SMARCA4-DTS	Young adults with a heavy smoking history	Rhabdoid or poorly differentiated phenotype	Loss of expression of SMARCA4 and SMARCA2, focal or diffuse expression of at least 2 of the following markers: SOX2, CD34 or SALL4.		Median survival 6-7 months	Response to pembrolizumab (case report)
Leora W,2014 and Xue Y, 2019	SCCOHT	29 year old	dominant appearance of small, hyperchromatic cells with scant cytoplasm with or without a rhabdoid appearance	Loss of SMARCA4 (95%)	Somatic or germline SMARCA4 mutation (93%)	Very aggressive	Sensitive to CDK4/6 inhibitors

Esther H,2017	SMARCA4-deficient NSCLC	72 year old	Differentiated (glandular or squamous) histology	Loss of SMARCA4, TTF-1 negative			predictor of chemosensitivity to platinum-based chemotherapy.
Abbas A,2017 ;Xue Y,2019 and Naito T,2019	SMARCA4-deficient pulmonary adenocarcinoma	60 year old	Focal unequivocal glands, no squamous differentiation, rhabdoid appearance	Loss of SMARCA4, $CK7^{+}$/HepPar-1^{+}/$TTF1^{-}$	no EGFR mutation	synchronous distant metastases (77%)	Sensitive to CDK4/6 inhibitors. Nivolumab (case report) .
Shih-Chiang H ,2020	Gastric cancer with altered SMARCA4 expression	70 year old	Undifferentiated carcinoma/tubular or solid adenocarcinoma, with or without rhabdoid morphology	Lost/reduced/heterogeneous expression of SMARCA4	SMARCA4 mutation or ARID1A mutation	16.3 month (median OS)	

SDUS, SMARCA4-deficient undifferentiated uterine sarcoma; SMARCA4-DTS, SMARCA4-deficient thoracic sarcoma; SCCOHT, Small cell carcinoma of the ovary, hypercalcemic type; NSCLC, non-small cell lung cancer
