# Supplementary material for: Undifferentiated colonic neoplasm with SMARCA4 germline gene mutation and loss of SMARCA4 protein expression: a case report and literature review
Source: Diagn Pathol. 2021 Apr 9;16:30. doi: 10.1186/s13000-021-01091-6 (PMC8033741; doi:10.1186/s13000-021-01091-6)
Supplement: Supplementary file 2 — Additional file 2. [file 13000_2021_1091_MOESM2_ESM.pdf]

Tissue processing and genomic DNA extraction

Formalin-fixed paraffin-embedded (FFPE) tissue sections were evaluated for tumor cell content using hematoxylin and eosin (H&E) staining. Only samples with a tumor content of ⩾20% were eligible for subsequent analyses. FFPE tissue sections were placed in a 1.5 microcentrifuge tube and deparaffinized with mineral oil. Samples were incubated with lysis buffer and proteinase K at 56 ° C overnight until the tissue was completely digested. The lysate was subsequently incubated at 80 °C for 4 hours to reverse formaldehyde crosslinks. Genomic DNA was isolated from tissue samples using the ReliaPrep™ FFPE gDNA Miniprep System (Promega) and quantified using the Qubit™ dsDNA HS Assay Kit (Thermo Fisher Scientific) following the manufacturer's instructions.

Library preparation and targeted capture

DNA extracts (30-200 ng) were sheared to 250 bp fragments using an S220 focused-ultrasonicator (Covaris). Libraries were prepared using the KAPA Hyper Prep Kit (KAPA Biosystems) following the manufacturer's protocol. The concentration and size distribution of each library were determined using a Qubit 3.0 fluorometer (Thermo Fisher Scientific) and a LabChip GX Touch HT Analyzer (PerkinElmer) respectively.

For targeted capture, indexed libraries were subjected to probe-based hybridization with a customized NGS panel targeting 733 cancer-related genes and 500 selected MSI loci. Repetitive elements were filtered out from intronic baits according to the annotation by UCSC Genome RepeatMasker [1]. The xGen® Hybridization and Wash Kit (IDT) was employed for hybridization enrichment. Briefly, 500 ng indexed DNA libraries were pooled to obtain a total amount of 2 μg of DNA. The pooled DNA sample was then mixed with human cot DNA and xGen Universal Blockers-TS Mix and dried down in a SpeedVac system. The Hybridization Master Mix was added to the samples and incubated in a thermal cycler at 95℃ for 10 min, before being mixed and incubated with 4 μl of probes at 65℃ overnight. The target regions were captured following the manufacturer's instructions. The concentration and fragment size distribution of the final library were determined using a Qubit 3.0 fluorometer (Thermo Fisher Scientific) and a LabChip GX Touch HT Analyzer (PerkinElmer) respectively.

DNA sequencing, data processing, and variant calling

The captured libraries were loaded onto a NovaSeq 6000 platform (Illumina) for 100 bp paired-end sequencing with a mean sequencing depth of 989.Raw data of paired samples (an FFPE sample and its normal control) were mapped to the reference human genome hg19 using the Burrows-Wheeler Aligner (v0.7.12) [2]. PCR duplicate reads were removed and sequence metrics were collected using Picard (v1.130) and SAMtools (v1.1.19), respectively. Variant calling was performed only in the targeted regions. Somatic single nucleotide variants (SNVs) were detected using an in-house developed R package to execute a variant detection model based on binomial test. Local realignment was performed to detect indels. Variants were then filtered by their unique supporting read depth, strand bias, base quality as previously described [3]. All variants were then filtered using an automated false positive filtering pipeline to ensure sensitivity and specificity at an allele frequency (AF) of ⩾ 5%. Single-nucleotide polymorphism (SNPs) and indels were annotated by ANNOVAR against the

following databases: dbSNP (v138), 1000Genome and ESP6500 (population frequency > 0.015). Only missense, stop-gain, frameshift and non-frameshift indel mutations were kept. Copy number variations (CNVs) and gene rearrangements were detected as described previously [3].

References:

1. Karolchik, D., et al., *The UCSC Table Browser data retrieval tool.* Nucleic Acids Res, 2004. **32**(Database issue): p. D493-6.

2. Li, H. and R. Durbin, *Fast and accurate short read alignment with Burrows-Wheeler transform.* Bioinformatics, 2009. **25**(14): p. 1754-60.

3. Su, D., et al., *High performance of targeted next generation sequencing on variance detection in clinical tumor specimens in comparison with current conventional methods.* J Exp Clin Cancer Res, 2017. **36**(1): p. 121.
